# Surgical treatment for locally advanced lower third rectal cancer after neoadjuvent chemoradiation with capecitabine: prospective phase II trial

**DOI:** 10.1186/1477-7819-7-52

**Published:** 2009-06-09

**Authors:** Mostafa Abd Elwanis, Doaa W Maximous, Mohamed Ibrahim Elsayed, Nabiel NH Mikhail

**Affiliations:** 1 Department of Radiotherapy, South Egypt Cancer Institute, Assiut, Egypt; 2 Department of Surgical Oncology, South Egypt Cancer Institute, Assiut, Egypt; 3 Department of Biostatistics and Cancer Epidemiology, South Egypt Cancer Institute, Assiut, Egypt

## Abstract

**Introduction:**

Treatment of rectal cancer requires a multidisciplinary approach with standardized surgical, pathological and radiotherapeutic procedures. Sphincter preserving surgery for cancer of the lower rectum needs a long-course of neoadjuvant treatments to reduce tumor volume, to induce down-staging that increases circumferential resection margin, and to facilitate surgery.

**Aim:**

To evaluate the rate of anal sphincter preservation in low lying, resectable, locally advanced rectal cancer and the resectability rate in unresectable cases after neoadjuvent chemoradiation by oral Capecitabine.

**Patients and methods:**

This trial included 43 patients with low lying (4–7 cm from anal verge) locally advanced rectal cancer, of which 33 were resectable. All patients received preoperative concurrent chemoradiation (45 Gy/25 fractions over 5 weeks with oral capecitabine 825 mg/m^2 ^twice daily on radiotherapy days), followed after 4–6 weeks by total mesorectal excision technique.

**Results:**

Preoperative chemoradiation resulted in a complete pathologic response in 4 patients (9.3%; 95% CI 3–23.1) and an overall downstaging in 32 patients (74.4%; 95% CI 58.5–85). Sphincter sparing surgical procedures were done in 20 out of 43 patients (46.5%; 95% CI 31.5–62.2). The majority (75%) were of clinical T_3 _disease. Toxicity was moderate and required no treatment interruption. Grade II anemia occurred in 4 patients (9.3%, 95% CI 3–23.1), leucopenia in 2 patients (4.7%, 95% CI 0.8–17) and radiation dermatitis in 4 patients (9.3%, 95% CI 3–23.1) respectively.

**Conclusion:**

In patients with low lying, locally advanced rectal cancer, preoperative chemoradiation using oral capecitabine 825 mg/m^2^, twice a day on radiotherapy days, was tolerable and effective in downstaging and resulted in 46.5% anal sphincter preservation rate.

## Background

Management of rectal cancer requires multidisciplinary treatment with standardized surgical, pathological and radiotherapeutic procedures [1,2]. Preoperative chemoradiation is considered the preferred treatment option for locally advanced rectal cancer (LARC) to reduce the incidence of local recurrence. This option is based on the knowledge that irradiation before surgery is more dose- and cost-effective than postoperative irradiation and less toxic. [3-5]. Sphincter preserving surgery for cancer of the lower rectum needs a long-course of neoadjuvant treatments to reduce tumor volume, to induce downstaging that increases circumferential resection margin, and to facilitate surgery [6].

Capecitabine is a fluoropyrimidine carbamate designed to generate 5-flurouracil (5-FU) preferentially in tumor cells as concentration of the key enzyme thymidine phosphorylase is higher in tumor cells compared with normal tissue. In preclinical studies, irradiation with thymidine phosphorylase was found to be upregulated in tumor tissue resulting in a supra-additive effect of capecitabine on radiotherapy [7-9]. Capecitabine is administered daily to mimic a continuous infusion of 5-FU [10].

Beyond the increase in convenience of using oral agents, data from phase II studies showed that the combination of preoperative capecitabine and radiotherapy in patients with LARC has significant anti-tumor activity, efficacy, and a low toxicity profile. These trials provide a clear rationale for replacing infusional 5-FU with oral capecitabine as part of chemoradiation [11,12].

## Patients and methods

This phase II trial included 43 patients and was conducted from March 2006 to September 2008 at Surgical Oncology Department and Radiotherapy Department, South Egypt Cancer Institute, Assiut University, Egypt. This includes all new patients admitted to these departments during this period.

All patients underwent a complete clinical examination, including digital rectal examination (DRE), a chest X-ray, an abdomino-pelvic computed tomography (CT), a transrectal ultrasound (TRUS) and a colonoscopy. Laboratory studies include: complete blood count (CBC), kidney function tests, complete liver functions, random blood sugar, and coagulation profile. All patients underwent treatment with curative intent for a histologically confirmed adenocarcinoma of the rectum. Informed consent was taken from the patients and the study was approved by the institutional ethics committee.

### Inclusion criteria

• Patients with lower third rectal carcinoma (within 4–7 cm of the anal verge using colonoscopy) with no clinical evidence of distant metastases

• Patients with T3–T4, N0 – N1 disease

• Patients with a performance status ≤ 2 according to the Eastern Cooperative Oncology Group (ECOG) system.

### Exclusion criteria

• Previous pelvic irradiation therapy

• Previous history of malignant disease

• Any other serious illness and/or major organ dysfunction

• Pregnancy or lactation.

Primary endpoints were grade of tumor downstaging and rate of sphincter preservation. Downstaging was assessed by comparing clinical stage and postchemoradiation pathologic stage. Secondary end points were toxicity and postoperative complications. Toxicity was scored according to Radiation Therapy Oncology Group (RTOG) criteria.

### Treatment details

#### Preoperative combined chemoradiation

##### Radiotherapy

• **Target volume: **included the rectum and the draining lymph node chains (pararectal, hypogastric, presacral lymph nodes) and was defined using the simulator. Target volume was localized with the patient in the prone position with a full bladder (to displace the small bowel anteriorly and superiorly). In the simulator, barium was introduced into the rectum and a wire marker was placed on the anal margin. Postero-anterior and lateral simulator films were taken. The contour of the pelvis was marked on the CT transverse section. The superior border of the target volume was placed at L5/S1 interspace, the inferior border at or distal to the obturator foramen and the lateral borders 1.5 cm outside the true bony pelvis. The anterior border was placed behind the symphysis pubis and the posterior border was placed 2 cm behind the sacrum.

• **Field arrangement**: Three field techniques were used (one posterior and two opposing wedged lateral fields) to give a homogeneous distribution to the target volume.

• **Dose and energy**: All patients were treated by a photon beam of either 6 or 15 MeV generated from a linear accelerator (Siemens Mevatron). The dose was 45 Gray in 25 fractions over 5 weeks prescribed at the isocenter of the plan according to ICRU report No. 50.

##### Chemotherapy

Capecitabine was administered orally at a dose of 825 mg/m^2 ^twice a day only on radiotherapy days. The first daily dose was given two hours before radiotherapy and the second dose twelve hours later. Dose modifications were applied if the patient experienced any grade 3 or 4 haematological toxicity or any grade 3 nonhaematological toxicity, such as hand-foot syndrome, except for alopecia.

##### Evaluation

Approximately 4–6 weeks after completion of chemo-radiation, physical examination, laboratory investigation and radiological studies (including CT scan of pelvis and abdomen) were preformed to evaluate patients for the possibility of surgery.

##### Surgical procedure

According to the protocol of the study, surgery was performed 4–6 weeks after the end of preoperative chemoradiation. All operations were performed by a total mesorectal excision technique which involves en-bloc resection of the rectum, perirectal fat and lymphoid tissue [13].

The decision to perform a low anterior resection versus an abdominoperinal resection depended on the distance of the lesion (after preoperative chemoradiation) from the anal verge and whether there was sphincter infiltration or not.

After histopathologic examination of the surgical specimens, rectal cancer regression grade (RCRG) was assessed, according to Wheeler et al. (2004) [14]. The three grades of RCRG are

• RCRG 1: Sterilization or only microscopic foci of adenocarcinoma with marked fibrosis

• RCRG 2: Marked fibrosis but macroscopic disease present.

• RCRG 3: Little or no fibrosis with abundant macroscopic disease.

### Statistical methods

Kaplan-Meier estimates of survival and local recurrence free survival were estimated.

## Results

Forty three patients with a lower third rectal cancer were included at this study; median age was 65 years old. Twenty eight patients were males and 15 patients were females with male to female ratio of 1.9: 1. Patients with T_4_N_1 _rectal cancer were considered unresectable (by pelvic CT scan) at presentation due to infiltration of the distal sacrum (in 3 patients), bladder neck infiltration (in 4 patients) and vaginal infiltration (in 3 patients). All patients and tumor characteristics are summarized in table 1.

**Table 1:** Patient and tumor characteristics

**Age:**	
Median	65
Range	36–73
**Gender, No. (%):**	
Male	28 (65.1)
Female	15 (34.9)

**Clinical tumor stage, No. (%):**	
T_3_N_0_	5 (11.6)
T_3_N_1_	15 (34.9)
T_4_N_0_	13 (30.2)
T_4_N_1_	10 (23.3)

**Performance status, No. (%)**	
0	33 (76.7)
1	10 (23.3)

**Distance from anal verge:**	
Median (cm)	5.5
0 – <5 cm, No. (%)	16 (37.2)
5 – ≤ 7 cm, No. (%)	27 (62.8)

Evaluation of the response to preoperative chemoradiation was presented in table 2. A complete pathologic response was found in 4 patients (9.3%, 95% CI 3–23.1). Overall downstaging was achieved in 32 patients (74.4%, 95% CI 58.5–85). No tumor progression had been observed. Postoperative pathologic assessment showed that T_0 _disease was observed in 4 patients (9.3%), T_1 _in 11 patients (25.6%), T_2 _in 8 patients (18.6%), T_3 _in 11 patients (25.6%), and T_4 _in 9 patients (20.9%).

**Table 2:** Distribution of clinical tumor stage compared with postchemoradiation pathologic stage.

Clinical staging	Postchemoradiotherapy pathologic (yp) staging									Total (%)
		
	ypT_0_N_0_	ypT_1_N_0_	ypT_1_N_1_	ypT_2_N_0_	ypT_2_N_1_	ypT_3_N_0_	ypT_3_N_1_	ypT_4_N_0_	ypT_4_N_1_	
c T_3 _N_0_	4	1	0	0	0	0	0	0	0	5 (11.6)

c T_3 _N_1_	0	4	6	0	3	0	2	0	0	15 (34.9)

c T_4 _N_0_	0	0	0	5	0	3	0	5	0	13 (30.2)

c T_4 _N_1_	0	0	0	0	0	0	6	0	4	10 (23.3)

Total (%)	4 (9.3)	5 (11.6)	6 (14)	5 (11.6)	3 (7)	3 (7)	8 (18.6)	5 (11.6)	4 (9.3)	43 (100)

The majority of patients (26 patients; 60.5%), were classified as RCRG2 (table 3), while only 4 patients (9.3%) showed RCRG1, and 13 patients (30.2%) showed RCRG3. Seven out of 11 patients (64%) with ypT_3 _tumor were classified as RCRG2.

**Table 3:** Pathologic T stage compared with RCRG following chemoradiation

RCRG	Postchemoradiotherapy pathologic (yp) staging					Total	
	
	ypT_0_	ypT_1_	ypT_2_	ypT_3_	ypT_4_	No.	% (CI)
1	4	0	0	0	0	4	9.3 (3.0–23.1)

2	0	11	8	7	0	26	60.5 (44.5–74.7)

3	0	0	0	4	9	13	30.2 (17.6–46.3)

Total (%)	4 (9.3)	11 (25.6)	8 (18.6)	11 (25.6)	9 (20.9)	43	100

**Figure 1:**
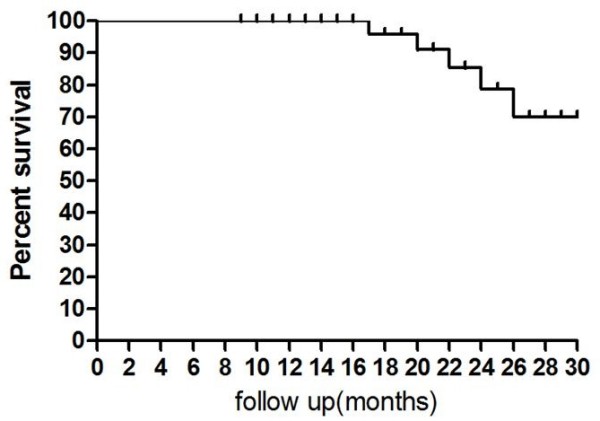
**The two year overall survival**. NB. After a median follow up of 25 months, the 2 year survival was 79%.

**Figure 2:**
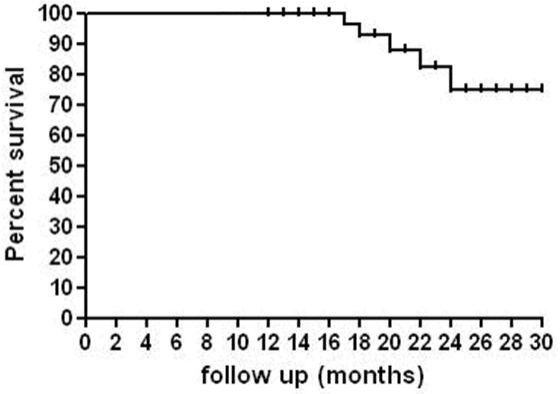
**The two year recurrence free survival**. NB. The 2 year recurrence free survival rate was 75%.

The overall sphincter preservation rate in the present study was 46.5% (20 out of 43 patients; 95% CI 31.5–62.2). Sphincter sparing surgical procedures were done in the majority of patients with clinical T_3 _rectal cancer (15 out of 20 patients; 75%), and in only 5 out of 23 (21.7%) patients with clinical T_4 _disease, 19 patients underwent abdominoperineal resection. Four patients with c T_4_N_1 _rectal cancer, showed no response to chemoradiation (ypT_4_N_1 _disease) and were considered unresectable during exploration because of tumor infiltration of the distal sacrum (in one patient), and presence of peritoneal deposits (in 3 patients). In those patients, palliative colostomy was done and biopsies from primary tumor and perirectal lymph nodes were taken. Sphincter preservation was achieved in the majority of patients with an initial tumor located 5 – ≤ 10 cm from anal verge (15 out of 27 patients; 55.6%) and in only 5 out of 16 patients (31.3%) with tumor located <5 cm. After a median follow up of 25 months (range 12–30 months), the 2 year overall survival was 79% (Fig. 1). There was only one patient out of 39 patients who underwent surgical resection due to local recurrence (2.5%), 18 months after treatment. This patient was reoperated for surgical resection of the recurrent tumor. The 2 year recurrence free survival rate was 75% (Fig. 2).

Toxicity was moderate and summarized in table 4. No hematological grade 3 or 4 toxicities occurred. Hematological toxicity was mild with grade II anemia in 4 patients (9.3%, 95% CI 3–23), and grade II leucopenia in 2 patients (4.7%, 95% CI 0.8–17). Regarding non hematological toxicity; hand-foot syndrome occurred only in one patient at the end of the treatment and required no treatment interruption. Grade II radiation dermatitis and diarrhea occurred in 4 patients (9.3%, 95% CI 3–23.1) and one patient (2.3%, 95% CI 0.1–13.8), respectively.

**Table 4:** Acute toxicity of preoperative chemoradiation

Toxicity	Grade I		Grade II	
	
	No	(CI)%	No	(CI)%
Anemia			4	9.3 (3.0–23.1)

Thrombocytopenia	10	23.3 (12.3–39.0)		

Leucopenia	10	23.3 (12.3–39.0)	2	4.7 (0.8–17.1)

Hand-Foot syndrome	1	2.3 (0.1–13.8)		

Radiation dermatitis	8	18.6 (8.9–33.9)	4	9.3 (3.0–23.1)

Nausea and vomiting	3	7.0 (1.8–20.2)		

Diarrhea	5	11.6 (4.3–25.9)	1	2.3 (0.1–13.8)

After completion of chemoradiation, sphincter conserving surgery was successfully performed in 20 patients (LAR in 19 patients and CAA in one patient, using hand-sewn technique in 14 patients and staplers in 6 patients, (due to short distal segment in 5 patients with lesions <5 cm from anal verge and narrow pelvis in one patient with lesions located 5–<10 cm from anal verge). Because of diabetes mellitus and atherosclerotic changes of mesenteric arteries, protective proximal stoma (colostomy) was applied in 3 patients. Nineteen patients underwent abdominoperineal resection. The circumferential resection margin was free in all cases. The postoperative 30 day mortality was not observed. Postoperative morbidity (10%) was in the form of anastomotic leakage (in one out of 20 patients; 5%), which healed conservatively and severe chest infection in one out of 20 patients; 5% (who underwent sphincter sparing surgery) and improved with broad spectrum antibiotics.

## Discussion

The gold standard treatment for locally advanced rectal cancer is neoadjuvant chemoradiotherapy using 5-FU [15]. Because of the short half-life of 5-FU in plasma, it should be given during the course of fractionated radiotherapy in the form of prolonged intravenous infusions which is better tolerated than a bolus administration [16,17]. However, capecitabine (an oral fluoropyrimidine that mimics continuously infused 5-FU) can replace intravenous 5-FU and may further enhance efficacy and tolerability. Capecitabine generates 5-FU preferentially at the tumor site by increasing the higher activity of the enzyme thymidine phosphorylase in tumor tissue compared with the healthy tissue [7]. Therefore, exposure of normal tissues to 5-FU within the radiation field is likely to be lower with oral capecitabine compared with intravenous 5-FU. Capecitabine also has proven activity as both adjuvant and first line treatment for colorectal cancer. The results from two large, randomized phase III trials including over 1200 patients showed that oral capecitabine was more active than bolus 5-FU/LV in terms of tumor response (26% versus 17%), and produced at least equivalent time to disease progression and overall survival [11].

In a study on cost effectiveness for preoperative staging of rectal cancer,[18] evaluation with abdominal CT plus endorectal ultrasound (EUS) was found to be the most cost-effective approach compared with abdominal CT plus pelvic MRI and CT alone. Because EUS can delineate the layers of the rectal wall, it is superior to CT in staging accuracy. EUS and MRI can be used as complementary methods in the preoperative staging of rectal cancer. EUS is more accurate in determining bowel wall penetration of the tumor, while MRI is comparable to EUS in the evaluation of lymph node involvement [19,20]. In the present study, the preoperative staging of rectal cancer was done by using abdominopelvic CT scan and EUS.

In light of the proven efficacy and safety benefits of the oral fluoropyrimidine capecitabine over bolus intravenous 5-FU/LV in the treatment of metastatic colorectal cancer and early stage colon cancer, a number of studies are evaluating capecitabine as a replacement for 5-FU/LV in chemoradiation schedules for patients with rectal cancer, (table 5). Beyond the increase in convenience of using oral agents, there is also a clear rationale for improved efficacy, and a reduction in the toxicity associated with radiotherapy [11].

**Table 5:** Phase II studies of capecitabine chemoradiation regimens in patients with LARC

**Reference**	**No. of evaluated patients**	**Treatment**	**Downstaging rate (%)**	**Response (%)**	**Sphincter preservation (%)**	**Main adverse events**
De Bruine et al [10]	60	Pelvic RT(2 Gy/day, total 50 Gy) +C (825 mg/m2 b.i.d. on radiotherapy days) ×5 weeks	67	pCR (13)	50	Grade 3 diarrhea (2%), radiation dermatitis (3%).

De Paoli et al [21]	53	Pelvic RT (1.8 Gy/day, total 45 Gy) + presacral boost(3 × 1.8 Gy) + C (825 mg/m2 b.i.d.), 7-days/week	57	pCR (24)	59	Grade 3 leucopenia (4%), hand-foot syndrome (4%).

Dunst et al [22]	69 (efficacy) 63 (safety)	Pelvic RT (1.8 Gy/day) + presacral boost(3 × 1.8 Gy) + C (825 mg/m2 b.i.d.), ×6 weeks	73	pCR (4)	NR	Grade 3 leuko-/lymphocytopenia (10%), diarrhea (4%),

Dunst et al [23]	96	Pelvic RT (50.4–55.8 Gy, conventional fractionation) + C (825 mg/m2 b.i.d.)	61	pCR **(7)**	51	Grade 3 lymphopenia (12%), leucopenia (16%), hand-foot syndrome (12%), diarrhea(7%)

Dupuis et al [24]	51	Pelvic RT (1.8 Gy/day, total 45 Gy) + C (825 mg/m2 b.i.d.), 7-days/week	58	pCR (20)	58	Grade 3 diarrhea (12%), radiation dermatitis (8%).

Kim et al [25]	38	Pelvic RT (1.8 Gy/day) + presacral boost(3 × 1.8 Gy) + C (825 mg/m2 b.i.d.) + LV (20/m2/day) days 1–14, 2 cycles of 14 days.	63	pCR (31)	72	Grade 3 hand-foot syndrome (7%), diarrhoea (4%), dermatitis (2%).

Lin et al [26]	53 (efficacy) 52 (safety)	Pelvic RT (1.8 Gy/day, total45 Gy) + primary tumor/perirectal node RT(1.75 Gy/day, total 52.5 Gy) + C(825 mg/m2 b.i.d.) ×5 weeks	62	pCR (17)	NR	Grade 3 diarrhoea (13%), radiation dermatitis (6%)

Velenik et al [27]	**57**	Pelvic RT (1.8 Gy/day, total 45 Gy) + C (825 mg/m2 b.i.d.), 7-days/week	49	pCR (9)	65.5	Grade 3 dermatitis (34.5%), diarrhoea (3.6%),

Present study	43	Pelvic RT (1.8 Gy/day, total 45 Gy) +C (825 mg/m2 b.i.d. on radiotherapy days) ×5 weeks	74	pCR (9)	46.5	Grade 2 anemia(9%) leucopenia(5%), diarrhoea (4%) Grade 1 hand-foot syndrome (2%).

The comparison of initial diagnosis and pathological findings showed downstaging in 32 patients (74.4%), with pathologically determined complete response rate (ypT_0_N_0 _disease) of 9.3% (4 out of 43 patients). The downstaging rate in the present study is comparable to those in most of phase II studies of chemoradiation regimens where the downstaging rates ranged from 58% to 73% [10,21-26]. On the other hand, our figure is much higher than that reported by Velinik et al., [27] (49%). The main reason for the lower downstaging rate in the reported study could be the prolonged radiotherapy course in 45.5% of patients with treatment interruptions of 3 days or more introduced in 18% of them. The pathological complete response rate, ranged from 4 to 31% in most of the reported studies [10,21-27]. The pathological complete response rate in the current study (9%) is comparable with those found by Dunst et al, [22](4%), Dunst et al, [23](7%), and Velenik et al, [27] (9%). On the other hand, it is lower than that reported by De Bruin et al.[10](13%), De Paoli et al,[21] (24%), and Kim et al, [25](31%). The higher figures in the reported studies than that in the present study may be due to more favorable distribution of T stage [10,21] or the use of radiation boost to tumor as well as use of leucovorine in addition to capcitabine [25]. In the subgroup of our patients, with unresectable rectal cancer (10 patients with T_4_N_1 _disease), the resectability rate was 60% (6 out of 10 patients). The unresectable 4 cases were one patient with persistent sacral infiltration and 3 cases with peritoneal deposits diagnosed during exploration. The resectability rate in those patients is comparable with that found by Veditic et al.[28] (62%) and lower than that reported by Glimelius et al.[29](71%). The use of methotrexate, 5-FU and leucovorine as the chemotherapy regimen, in the reported study, may explain their higher figure of resectability rate [29].

In the present study, complete response to chemoradiotherapy resulted in favorable tumor and nodal stage. However, despite a complete regression of the mural tumor, the mesorectum can harbor residual cancer deposits. Therefore, even those in whom a complete response is suspected should undergo standard excisional surgery [30].

The sphincter preservation rate, in the present study, was 46.5% (20 out of 43 patients). The reported sphincter preservation rates ranged from 50% to 72% [10,21,23-25,27]. These higher rates in the reported studies than that in the current study, may be due to higher radiotherapy dose (50 Gy)[10,25], capcitabine administration throughout the radiotherapy period including weekends [21,23,24], the more favorable distribution of T stage,[25,27] or the use of leucovorine in addition to capcitabine in the chemotherapy regimen.[25]

Sphincter preservation was achieved in the majority of patients with initial tumor located 5 – ≤ 10 cm from anal verge (55.6%) and in only 5 out of 16 patients (31.3%) with tumor located <5 cm from anal verge. This is in agreement with De Bruin et al.[10] who found a sphincter preservation rate of 25% in patients with initial rectal cancer <5 cm from anal verge compared to 65% in tumors 5 – 10 cm from anal verge. However, many authors stated that the decision to perform radical surgery (APR) should not be changed even when downstaging occurs. Between 1999 and 2002, 316 patients from 19 institutions were enrolled. [31] The sphincter preservation rate was 61% in the 5 × 5 Gy radiotherapy arm and 58% in the radiochemotherapy arm, p = 0.57. Despite significant downsizing, chemoradiation did not result in increased sphincter preservation rate in comparison with short-term preoperative radiotherapy. The surgeons' decisions were subjective and based on pre-treatment tumour volume at least in clinical complete responders [31].

No hematological grade 3 or 4 toxicities occurred. Grade II anemia occurred in 9.3% and grade II leucopenia in 4.7% of patients. Hand-foot syndrome occurred only in one patient at the end of the treatment and required no treatment interruption. Grade II radiation dermatitis and diarrhea occurred in 9.3% and in 2.3% of patients respectively. The incidence of acute toxicity in the present study is obviously lower than other phase II trials using capcitabine, where 4–16% grade 3 leucopenia [21-23], 2% grade 4 anemia and neutropenia [32], 4–12% grade 3 hand-foot syndrome [21,23,25] and 2–13% grade 3 diarrhea [10,21-27] and 2–34.5%% grade 3 radiation dermatitis [10,24-27] were reported. These differences can be explained on the ground of use of presacral boost [21-23,25,26] and capecitabine administration. In the present study, because of the two-day resting period every 5 days (i.e. capecitabine was given on radiation days only), toxicity might therefore be lower than that in the reported series, where capecitabine was administered twice daily, seven days a week.[10,21-27]

## Conclusion

Despite statements by other authors that chemoradiation did not result in increase sphincter preservation rate, our study showed a 74.4% downstaging rate and 46.5% sphincter preservation rate. Therefore, preoperative chemoradiation using capecitabine (825 mg/m^2 ^twice a day) may be applied to patients with locally advanced low lying rectal cancer for downstaging and facilitate sphincter preservative surgery. Further studies with longer follow up period are recommended to assess the effect of this protocol on survival.

## Consent

Written informed consent was obtained from all patients. Institutional approval was taken before the start of this study.

## Competing interests

The authors declare that they have no competing interests.

## Authors' contributions

MA participated in design and coordination of chemoradiation; DWM participated in the design, carried out operative intervention and shared in the manuscript writing; MIE participated in design, coordination of chemoradiation and shared in the manuscript writing; NNHM participated in follow up of the patients and the manuscript writing.

All authors read and approved the final manuscript.
